# Epistasis from functional dependence of fitness on underlying traits

**DOI:** 10.1098/rspb.2012.1449

**Published:** 2012-08-15

**Authors:** Hsuan-Chao Chiu, Christopher J. Marx, Daniel Segrè

**Affiliations:** 1 Bioinformatics Program, Boston University, Boston, MA 02215, USA; 2 Department of Biology and Department of Biomedical Engineering, Boston University, Boston, MA 02215, USA; 3 Department of Organismic and Evolutionary Biology and Faculty of Arts and Sciences Center for Systems Biology, Harvard University, Cambridge, MA 02138, USA

**Keywords:** epistasis, benefit–cost model, evolution, pleiotropy, modularity

## Abstract

Epistasis between mutations in two genes is thought to reflect an interdependence of their functions. While sometimes epistasis is predictable using mechanistic models, its roots seem, in general, hidden in the complex architecture of biological networks. Here, we ask how epistasis can be quantified based on the mathematical dependence of a system-level trait (e.g. fitness) on lower-level traits (e.g. molecular or cellular properties). We first focus on a model in which fitness is the difference between a benefit and a cost trait, both pleiotropically affected by mutations. We show that despite its simplicity, this model can be used to analytically predict certain properties of the ensuing distribution of epistasis, such as a global negative bias, resulting in antagonism between beneficial mutations, and synergism between deleterious ones. We next extend these ideas to derive a general expression for epistasis given an arbitrary functional dependence of fitness on other traits. This expression demonstrates how epistasis relative to fitness can emerge despite the absence of epistasis relative to lower level traits, leading to a formalization of the concept of independence between biological processes. Our results suggest that epistasis may be largely shaped by the pervasiveness of pleiotropic effects and modular organization in biological networks.

## 1. Introduction

Epistasis describes a fundamental nonlinearity in biological systems, capturing the fact that the phenotypic effect of a genetic mutation or allele variant could depend on another mutation. Epistasis is suggested to play an important role in evolutionary dynamics, e.g. by shaping the fitness landscape [1,2], maintaining sexual reproduction [3,4] and affecting the speed of adaptation [5–8]. Large-scale systematic studies of single and double gene deletions have also revealed that epistasis is useful in understanding the organization of living systems into modules, as genes belonging to the same biological process tend to share similar profiles of epistatic interactions with other genes [9,10]. Given the influence of epistasis on evolutionary processes, and given the modular structure of epistatic interaction networks, one may wonder whether deeper insights can be obtained on how the modular organization of biological networks affects (and is affected by) epistasis and adaptation. The connection between modularity and evolution has been addressed from multiple independent standpoints [11–16]. Yet few concepts seem to be more appropriate than epistasis for trying to relate the architecture of biological networks with the evolutionary forces that gave rise to such networks. This is ultimately due to the fact that epistasis captures in an elementary way the complexity of the genotype–phenotype map.

For quantitative traits, epistasis can be analysed mathematically. Upon a double mutation, a given quantitative trait could turn out to be higher or lower than expected based on individual mutations, leading to a quantifiable positive or a negative epistasis, respectively. Special caution should be used in comparing the definitions of epistasis across different research areas, as multiple, sometime conflicting, terminologies have been adopted. A commonly used classification is the one between synergistic and antagonistic epistasis. Synergistic (antagonistic) epistasis occurs when the joint effect of two alleles is more (less) severe than expected. Note that, based on the terminology adopted here, negative epistasis between deleterious mutations corresponds to synergism, while negative epistasis between beneficial mutations is classified as antagonism (table 1). In addition, the very definition of the baseline expectation relative to which epistasis is quantified is still the subject of active debate [17–20]. In this work, we assume that mutational effects combine multiplicatively in the absence of epistasis (see also §2).

**Table 1.** Definitions and conventions for epistasis in the current work. We quantify the degree of epistasis (*ɛ*) as the deviation of the joint effect of mutations from the expectation in multiplicative scale (see equation (2.1)). Synergistic epistasis occurs when the joint effect of two alleles is reinforced (e.g. more severe than the multiplicative expectation), while antagonistic epistasis happens when the joint effect is buffered (less severe than the multiplicative expectation) by the interaction between alleles. As illustrated in the table, negative epistasis (*ɛ* < 0) may point to synergistic or antagonistic behaviour based on whether the mutations are both beneficial or both deleterious.

	type of mutations	
	deleterious	beneficial
synergistic	*ɛ* < 0	*ɛ* > 0
antagonistic	*ɛ* > 0	*ɛ* < 0

The role of epistasis in evolution constitutes a particularly important and debated question [21], which involves understanding the sources and the consequences of the average and variance of the distribution of epistatic effects [22]. Therefore, considerable effort has been put into elucidating the distribution of epistasis, both from a theoretical and from an experimental perspective [23,24]. Some early experimental studies, partially motivated by the possible relevance of synergistic effects between deleterious mutations in the evolution of sex [3,4], had found nearly symmetric distributions of epistasis [25–28]. Subsequent large-scale studies under different proportions of beneficial versus deleterious mutations have reported both negative [10] and positive [29] epistasis trends, leaving the problem fundamentally unsettled. From a theoretical perspective, models of fitness landscapes have been used for providing potential explanations for observed epistasis trends. Antagonism between beneficial mutations, for example, can be predicted by a fitness landscape model that assumes ubiquity of stabilizing selection [30]. In addition, stoichiometric models of metabolic networks have been useful in exploring the distribution and network organization of gene–gene interactions in metabolism [9,31], and in providing mechanistic explanations for measured distributions [29,32]. However, none of these prior theoretical works seems to have explicitly addressed the question of how epistasis quantitatively depends on the modular organization of the genotype–phenotype mapping, and on the degree of pleiotropy.

A hint to how modularity and epistasis relate to each other was recently offered by an experimental evolution study that identified a diminishing returns trend among pairs of beneficial alleles [7] (see also recent studies [8,33]). This study reported antagonistic epistasis between beneficial alleles that arose during 600 generations of evolution of an engineered strain of *Methylobacterium extorquens*. It was found that the observed beneficial alleles improve fitness either by enhancing metabolic capacity or by alleviating protein expression-related costs. This observation suggested that microbial fitness could very coarsely be treated as a modularly structured function of two separate phenotypic traits, a metabolic benefit and a protein expression cost. In fact, a simple benefit–cost model was sufficient to quantitatively predict fitness values for multi-allele strains, and to quantitatively recapitulate the antagonistic trend for epistasis between genes in different loci of the genome [7]. Benefit–cost models had been used to describe fitness in previous studies of evolutionary adaptation, ranging from rapid adaptation of protein expression in new environments [14], to optimal regulatory design upon biochemical noise [34] and optimal transcriptional regulation of metabolism [35].

Here, inspired by the previously demonstrated relevance of benefit–cost models in evolutionary research, we use a benefit–cost model as a starting point for quantifying epistasis based on the dependence of fitness on multiple traits. In particular, we first extend the benefit–cost model from a way to explain an observed case of antagonism between beneficial mutations [7], to a general statistical analysis of expected epistasis distributions among mutations with a given chance of being beneficial or deleterious. In the second part of our work, we generalize these results to show that the degree of epistasis can be estimated analytically for an arbitrary dependence of fitness on simpler quantitative traits, providing a new mathematical link between epistasis, pleiotropy and modularity. Note that our analysis is mostly focused on analysing the interactions between two mutations, without delving into the problem of how multiple perturbations accumulate. In addition, we restrict our calculations almost entirely to a fitness function that depends on two traits (also previously called two-dimensional epistasis [36]), leaving possible extensions to multidimensional epistasis [36] as a topic for potential follow-up research.

## 2. Background and definitions

**Figure 1.**
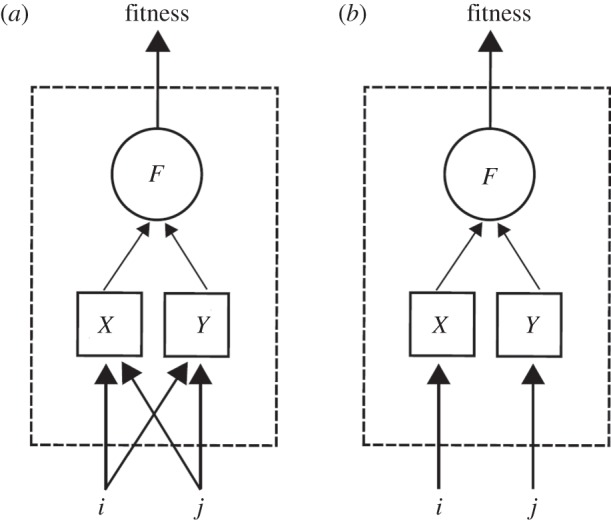
Schematic depiction of how we quantify epistasis relative to a fitness function that depends on two quantitative traits, or phenotypes. (*a*) Two alleles or genetic perturbations *i* and *j* are assumed to potentially affect multiple traits, here *X* and *Y* (‘low-level traits’). The phenomenon in which a genetic perturbation affects multiple traits is called pleiotropy. Here we assume that there is no epistasis at the level of the individual traits *X* and *Y*. A ‘high-level trait’ (e.g. fitness *f*) is defined as a function *F* of the two traits *X* and *Y*. These assumptions allow us to predict how the functional shape of *F* affects epistasis between the two perturbations. Without any knowledge of this internal structure (dashed box), the presence of epistasis could only be measured experimentally, but not inferred mathematically. (*b*) The same model as described above, in the absence of pleiotropy. In this case, perturbations *i* and *j* affect each a single trait, i.e. *X* and *Y* respectively, and can be thought of acting on different modules. Depending on the function *F*, this may still lead to epistasis.

The main premise of the current work is that a high-level trait, or phenotype, such as fitness (*f*) can be phenomenologically expressed as a function *F* of two basic observable traits, *X* and *Y*: *f* = *F*(*X*,*Y*). For example, the growth rate of a bacterium may be expressed as a function of its respiratory and fermenting capacities [37]. In addition to a wild-type organism (e.g. a bacterial strain), we consider two mutant strains with genetic modifications at loci *i* and *j*, respectively, and a double mutant strain which has both *i* and *j* alleles modified. For each of these four strains, we hypothesize that it is possible to independently measure the overall fitness (*f*_0_ for the wild-type, *f_i_* and *f_j_* for the single mutants, and *f_ij_* for the double mutant), as well as each of the two basic traits *X* and *Y* (*x*_0_ for the wild-type, *x_i_* and *x_j_* for the single mutants, and *x_ij_* for the double mutant, etc.). The general question we are concerned with is whether we can estimate epistasis with regard to fitness between perturbations *i* and *j* given some assumptions on how these mutations affect phenotypes *X* and *Y*, and given the functional dependence of *F* on *X* and *Y* (figure 1).

In the absence of epistasis, it is often assumed that mutational effects should combine multiplicatively, i.e. *f_ij_*/*f*_0_ *=* (*f_i_*/*f*_0_)(*f_j_*/*f*_0_) ([7–10,25,31,38], see also discussion on alternative definitions [39]). In other words, under this assumption, absence of epistasis means that perturbations will tend to affect fitness in a way that is proportional to the fitness itself, and independent of the genetic background (as easily seen by rewriting the previous expression as *f_ij_*/*f_j_* *= f_i_*/*f*_0_). Epistasis can be therefore seen as a deviation from this null multiplicative expression and quantified as2.1
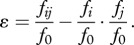


This equation has been used previously [7–10,25,31,38] for assessing the degree of epistasis, given experimentally measured or computationally predicted fitness values for the wild-type and mutant strains. It is through this equation that one can determine whether epistasis is positive (*ɛ > 0*) or negative (*ɛ < 0*). In this work, we will use this equation to determine how an analytical expression for fitness as a function of other measurable traits (and of the mutations that affect such traits), translates into a prediction of epistasis. We will restrict our analysis to the simplest case in which alleles exhibit no direct epistasis with regard to either of the two underlying traits *X* and *Y* (i.e. *x_ij_*/*x*_0_ *=* (*x_i_*/*x*_0_)(*x_j_*/*x*_0_) and *y_ij_*/*y*_0_ *=* (*y_i_*/*y*_0_)(*y_j_*/*y*_0_)). Under this assumption, epistasis relative to fitness emerges only as a consequence of the functional dependence of *F* on *X* and *Y* (figure 1).

The benefit–cost model used to explain an antagonistic epistasis pattern that emerged during adaptation of an *M. extorquens* strain [7] constitutes a special case of the *F* function introduced earlier. In this case, in analogy to Dekel & Alon [14], *X* = *b* is the growth advantage (benefit), and *Y* = *c* is the burden (cost) associated with the operation of the biological network, and *F = X* − *Y = b* – *c*. In this model, fitness of the wild-type strain can be written as the difference between a basal benefit and a basal cost term:2.2
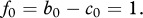


The quantities *f*_0_ and *c*_0_ can be experimentally measured to operationally define the basal benefit *b*_0_ as demonstrated in earlier studies [7,14]. A mutant allele *i* is assumed to pleiotropically alter *b*_0_ or *c*_0_ with factors *λ_i_* and *θ_i_* respectively, yielding a fitness:2.3
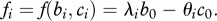
To avoid confusion, it is important to stress that *λ_i_* represents the effect of a single mutation (*i*) on the benefit component of the fitness (if *i* has no effect on the benefit, then *λ_i_* = 1), and is not proportional to the number of mutations accumulated in a longer-term evolutionary process. Rather, multiple mutations are assumed to act by the action of further multiplicative factors. For example, a successive allele *j*, on the background of *i*, is assumed again to act multiplicatively on the benefit and cost components, giving rise to the fitness of the double mutant2.4
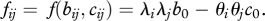


Note that the changes in each trait are not simply given by the *λ* and *θ* factors, but rather also include a dependence on the unperturbed trait, e.g. *Δb = *λ*_i_b*_0_ − *b*_0_ *=* (*λ_i_ − 1*)*b*_0_. In our previous work [7], experimentally determined values of *b*_0_, *c*_0_, and of *λ_i_* and *θ_i_* for each allele, were used in equation (2.4) (and its extension to more than two mutations) to provide accurate predictions of the fitness of multi-allele strains, and to explain the observed antagonistic epistasis among some beneficial alleles. Here instead, we explore the space of possible pairs of mutational effects to infer statistical properties of epistasis.

## 3. Results

### (a) An analytical expression of epistasis in the benefit–cost model

Our first goal is to determine analytically the magnitude and sign of epistasis relative to fitness under the assumptions of the benefit–cost model. As shown in detail in electronic supplementary material, §B, this can be achieved by substituting the expressions for the fitness of single and double mutants (equations (2.3) and (2.4)) into the definition of epistasis (equation (2.1)), yielding:3.1
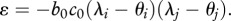


This is a remarkably simple expression, in which epistasis turns out to be computable as the product of a term that depends only on the unperturbed state parameters (*b*_0_, *c*_0_), and on a term that depends only on the phenotypic effects of the mutations (the *λ_i_, *θ*_i_, *λ*_j_* and *θ_j_* parameters). From equation (3.1), one can see that no epistasis ensues in the benefit–cost model if *λ* = *θ* for one or both mutations (see electronic supplementary material, figure S9 for additional clarifications, including an extension to more than two mutations). Importantly, given that the benefit and cost terms are defined as positive (*b*_0_ > 1 and *c*_0_ > 0), the sign of epistasis is entirely determined by the (*λ_i_* – *θ_i_*)(*λ_j_* – *θ_j_*) product. Hence, in this model, the sign of epistasis between two mutations depends only on their mutational effects on the benefit and the cost, but not on the initial unperturbed values. In addition, from equation (3.1), one can see that positive epistasis can be obtained only by combining mutations that have (*λ* – *θ*) values of opposite sign. Additional steps are required to determine how this expression for epistasis (and especially its sign) depends on whether individual mutations are beneficial or deleterious.

### (b) The benefit–cost model imposes a negative bias in the distribution of epistasis

Equation (3.1) predicts the degree of epistasis for specific combinations of two mutations. In this section, we show how this same equation can be also used to draw general conclusions about the expected distribution of epistasis between a pair of perturbations in the benefit–cost model. The fact that the sign of epistasis is dictated by the product of (*λ*–*θ*) terms suggests that a statistical analysis of how these terms are distributed could provide information about the distribution of epistasis itself.

**Figure 2.**
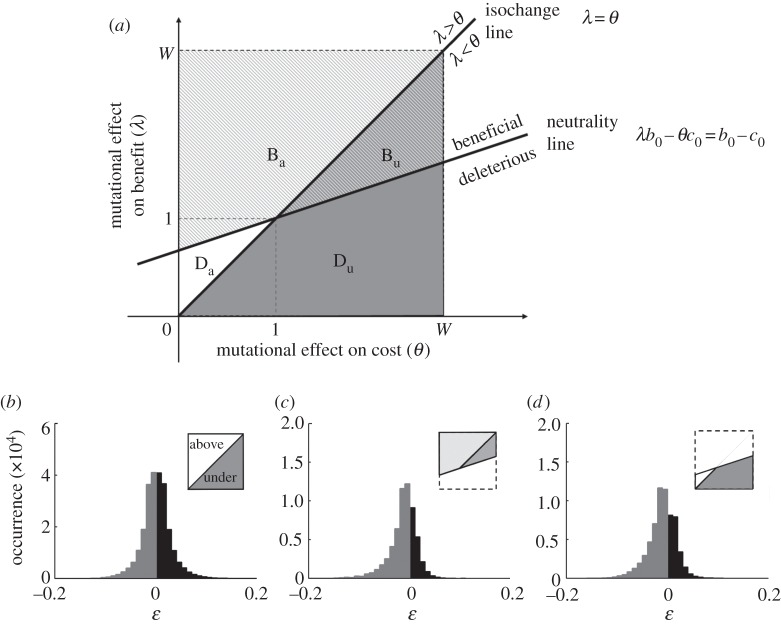
Estimating epistasis through a geometrical representation of perturbations in phenotype space. (*a*) The (*λ*,*θ*) plane, a geometrical representation of possible mutant alleles in a benefit–cost model of fitness. Any allele (e.g. *i*) can be represented as a point with coordinates (*λ_i_*,*θ_i_*) corresponding to the multiplicative alterations of the benefit and cost, respectively. We assume that both *λ* and *θ* can have values between zero and *W*. Throughout the paper, we assume *W* = 2, so that beneficial and deleterious mutations have equal chance of being chosen when sampling uniformly. The (*λ*,*θ*) plane is divided into four regions by the neutrality line (corresponding to mutants with fitness equal to the wild-type) and the isochange line (corresponding to mutations such that *λ_i_* = *θ_i_*). The intersection between these two lines (i.e. the point (*λ*,*θ*) = (1,1)) corresponds to the wild-type strain. *B*_a_ is the area containing beneficial alleles above the isochange line; *B*_u_ is the area containing beneficial alleles under the isochange line. *D*_a_ and *D*_u_ are similarly defined for deleterious alleles. The combination of two alleles both lying above the isochange line will give rise to negative *ɛ*, as evident from equation (3.1). In general, the sign of *ɛ* depends on the chance of selecting alleles from different regions in the (*λ*,*θ*) plane. The maximum value of *B*_u_ = (*W* − 1)^2^/2 occurs when the slope of the neutrality line is zero (*c*_0_ = 0). The corresponding *B*_a_ in this situation is *B*_u_ + (*W* − 1). When we increase the slope, *B*_u_ decreases (while *B*_a_ increases) monotonically as *c*_0_ goes up, until *B*_u_ reaches its minimum value at zero when *c*_0_ = *b*_0_ (slope of neutrality line = 1). Thus, it is always *B*_a_ > *B*_u_. (*b*) Without imposing any constraint on whether mutations are beneficial or deleterious the regions above and under the isochange line have equal chance to occur (inset), leading to an unbiased epistasis distribution. (*c*,*d*) Negative bias between strictly beneficial alleles (*c*, region *B*_a_ > *B*_u_ shaded in inset) and between strictly deleterious alleles (*d*, region *D*_u_ > *D*_a_ shaded in inset) can be demonstrated analytically, and is confirmed here by simulations (see the electronic supplementary material, §A).

To reason about this problem, one can visualize possible choices of perturbations on the (*λ*,*θ*) plane (figure 2). Each perturbation in the benefit–cost model can be represented as a point in this plane. The point of coordinates (1,1) corresponds to the wild-type, i.e. the unperturbed system. For simplicity, we assume here that *λ* and *θ* cannot exceed a given value *W*. Two fundamental lines can be drawn on this plane. One line, which we call the *isochange line*, is defined by the equation *λ* = *θ*, and corresponds to all individual perturbations that change both the benefit and the cost by the same multiplicative factor. Note that the isochange line does not have in itself an immediate interpretation in terms of epistasis (e.g. it does not represent a boundary between positive and negative epistasis); rather, as reasoned later, it is an abstract geometrical construct that will help us to determine the chance of observing a given sign of epistasis for two mutations, through equation (3.1). If we think of two mutations as two points with coordinates (*λ_i_*,*θ_i_*) and (*λ_j_*,*θ_j_*), the positions of these points relative to the *isochange line* will determine the signs of the terms (*λ_i_ − *θ*_i_*) and (*λ_j_ − *θ*_j_*), and hence, based on equation (3.1), the sign of epistasis between such mutations. If no other constraints exist in the system, upon uniformly sampling pairs of points in the (*λ,*θ**) plane (with *W* = 2), it is equally likely to choose positive or negative (*λ − *θ**) terms, giving rise to no obvious bias in the distribution of *ɛ* (figure 2*b*).

A second fundamental line in the (*λ*,*θ*) plane is the line that partitions beneficial from deleterious mutations. This line, which we call *neutrality line* (*λ_i_b*_0_ – *θ_i_c*_0_ *=* 1), corresponds to all possible choices of *λ* and *θ* whose combined effect is to leave fitness equal to the wild-type value of 1. All points above this line are associated with individual beneficial mutations; all points below this line correspond to individual deleterious mutations. It is now possible to ask whether any bias in the distribution of *ɛ* may be expected among strictly beneficial mutations. In other words, we ask whether a repeated uniform sampling of pairs of points from the region above the neutrality line will preferentially yield positive or negative values of *ɛ*, based on equation (3.1). Note that the sampling we are performing here is a sampling in phenotype space, and is not meant to provide insights into the actual rates of beneficial/deleterious mutations in the genome. What determines the sign of *ɛ*, for a given pair of beneficial perturbations, is whether points selected above the neutrality line fall above (area *B*_a_) or under (area *B*_u_) the *isochange line.* Specifically, as explained in table 2, it is easy to see that the difference in the chance to obtain a negative versus a positive *ɛ* is *p*(*ɛ* < 0) *− p*(*ɛ* > 0) = (*B*_a_ − *B*_u_)^2^/(*B*_a_ + *B*_u_)^2^. This expression is always positive as long as *B*_a_ ≠ *B*_u_, a condition geometrically confirmed by figure 2*.* Thus, under the benefit–cost model, one should expect a bias towards negative *ɛ* (antagonistic epistasis) among beneficial mutations (figure 2*c*), consistent with the specific observations of [7]. As shown in figure 2*d*, a similar result can be obtained for deleterious mutations (points below the neutrality line). In this case, again, one can infer a tendency towards negative *ɛ*, indicating a bias towards synergistic epistasis between deleterious mutations. It is possible to further generalize the earlier-mentioned results to perturbations with any given probability *ρ* of being beneficial (i.e. the fraction of beneficial mutations *ρ* can be any value other than 0.5). It can be shown (see the electronic supplementary material, §C) that in this general case, one can analytically compute the excess probability of negative epistasis (*Δp^*ɛ*^* = *p*(*ɛ* < 0) *− p*(*ɛ* > 0)) as a function of *ρ* and *c*_0_. In particular, under simplifying assumptions, one obtains *Δp^*ɛ*^ =* (2*ρ* − 1)^2^ × (2*c*_0_ + 1)^2^/(2*c*_0_ + 2)^2^ (see the electronic supplementary material, figure S2*b*). Unless *ρ =* 0.5, this expression is expected to be always positive, demonstrating that the negative bias in the distribution of epistasis is a general property of the benefit–cost model. We further support this analytical conclusion with sensitivity analyses against choices of *ρ* and *c*_0_. This negative bias is consistently obtained, with no substantial difference computationally (see the electronic supplementary material, figure S2*a*) or analytically (see the electronic supplementary material, figure S2*b*). In addition, no significant deviations from this trend are observed upon introducing an arbitrary interdependence between *λ* and *θ* in the form of a rotated Gaussian bivariate distribution (see the electronic supplementary material, figure S3).

**Table 2.** Contingency table for the phenotypic values of strictly beneficial alleles. The categories of *ɛ* classified by the four conditions are analogous to the possible outcomes of tossing a coin twice, and allow us to compute the overall probability of negative and of positive *ɛ*, giving 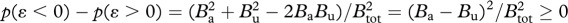, where *B*_tot_ = *B*_a_ + *B*_u_.

*ɛ*	−	−	+	+
condition	*λ_i_* > *θ_i_*	*λ_i_* < *θ_i_*	*λ_i_* > *θ_i_*	*λ_i_* < *θ*_i_
	*λ_j_* > *θ_j_*	*λ_i_* < *θ_j_*	*λ_j_* < *θ_j_*	*λ_j_* > *θ_j_*
region (*i* , *j*)	(*B*_a_, *B*_a_)	(*B*_u_, *B*_u_)	(*B*_a_, *B*_u_)	(*B*_u_, *B*_a_)
*p*(condition)	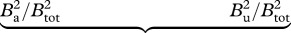		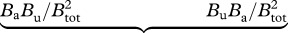	
	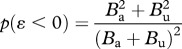		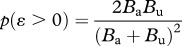	

**Figure 3.**
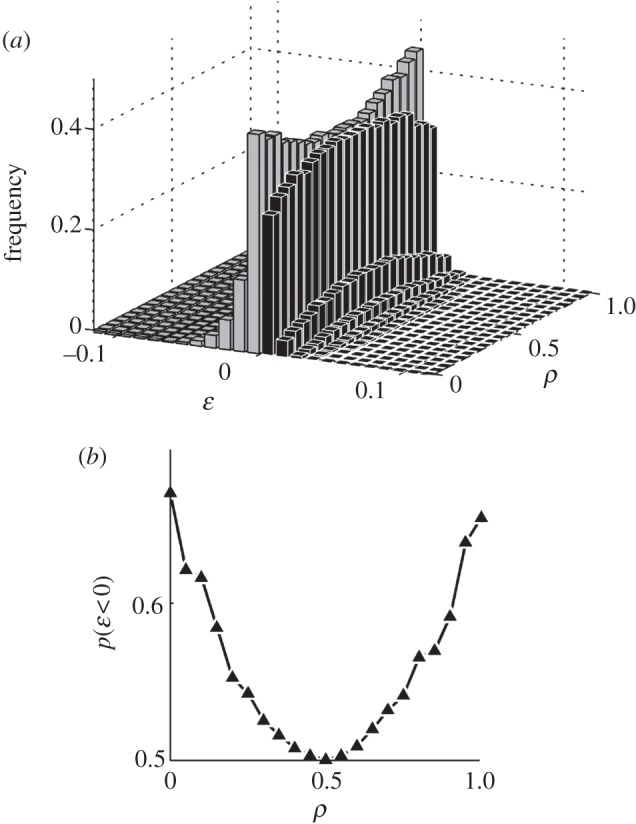
Numerically computed epistasis distributions show a generic negative trend for all possible proportions of beneficial mutations. Each bell-shaped histogram corresponds to the distribution of epistasis at a given fraction of beneficial mutations (*ρ*). For visual clarity, bars associated with negative *ɛ* are depicted in light grey, while bars for positive *ɛ* are depicted in dark grey. The front slice (*ρ* = 0) is the same distribution shown in figure 2*d*. (*a*) The concave shape for the negative *ɛ* bars across different values of *ρ* indicates that the bias towards negative *ɛ* increases as the portion of beneficial allele moves away from 0.5. (*b*) Negative epistasis is more likely to occur when the single mutants are dominated by mostly beneficial (*ρ* ≫ 0.5) of mostly deleterious alleles (*ρ* ≪ 0.5).

While the geometrical arguments illustrated earlier provide estimates of the bias in the distribution of *ɛ* as a function of the fraction, *ρ*, of beneficial mutations, they do not allow us to predict the shape of distribution itself. We used computer simulations (see the electronic supplementary material, §A) to explore the full spectrum of epistasis distribution as a function of *ρ*. Again, here *ρ* is a phenotypic measure; we make no assumption on the connection between random mutations and specific values of *ρ*. The simulations confirm that negative *ɛ* is more likely to occur over the whole range of *ρ* values (figure 3*a*). The bias (prevailing negative epistasis) reaches a maximum in both extreme cases (strictly beneficial or strictly deleterious; figure 2*c*,*d*) while it becomes less and less pronounced as *ρ* approaches 0.5 (figure 3*a*,*b*). Note that for distributions derived from combinations of beneficial and deleterious mutations, a negative *ɛ* cannot be easily associated with synergistic or antagonistic trends, as different pairs contributing to the distribution will have different effects (including sign epistasis, an interesting case where an allele is beneficial on some genetic backgrounds but deleterious on others; see electronic supplementary material, figure S7 for more details). Computer simulations also indicate that these epistasis trends are robust over a broad range of values for *c*_0_ (see the electronic supplementary material, figure S2), and that they would equally ensue in a more complex model involving multiple cost components (e.g. *f*_0_ = *b*_0_ − *c*_0_ − *d*_0_ − *e*_0_, electronic supplementary material, figure S1).

### (c) Analytical estimate of epistasis for an arbitrary function *F*

Most of our study so far has been focused on a specific dependence of fitness on two traits, i.e. the difference between a benefit and a cost trait. We next generalize our analysis to ask whether it is possible to estimate epistasis when fitness depends in an arbitrary way on two quantitative traits *X* and *Y*, e.g. it is the sum, the product or any arbitrary function *F* of such traits. As performed for the derivation of equation (3.1), we need to substitute the expressions for fitness of single and double mutant strains into equation (2.1) to compute *ɛ = F*(*x_ij_,y_ij_*)*/F*(*x*_0_,*y*_0_) *−* [*F*(*x_i_,y_i_*)*/F*(*x*_0_,*y*_0_)]*·*[*F*(*x_j_,y_j_*)*/F*(*x*_0_,*y*_0_)]. In this case, however, no further result can be obtained unless additional simplifying assumptions are made about the system. One possible such assumption is that mutations cause small perturbations to the traits (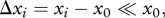 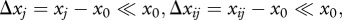 and similarly for *Δy*). Under this assumption, one can perform a Taylor expansion of each term in the above expression for *ɛ*, e.g. for *F*(*x_i_*,*y_i_*) = *F*(*x*_0_ + *Δx_i_*,*y*_0_ + *Δy_i_*). Note that because the product *F*(*x_i_,y_i_*) *· F*(*x_j_,y_j_*) will give rise to second order terms (e.g. in *Δx^2^*), it is essential to perform the Taylor expansion to the second order. In the derivation, presented in detail in electronic supplementary material, §D, we assumed (as for the benefit–cost model) that there is no epistasis between perturbations relative to each of the two traits *X* and *Y*. After some algebraic rearrangements, we obtained for epistasis3.2
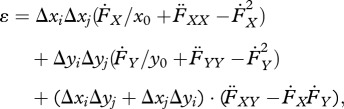
where 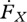, 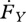, 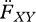, 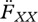 and 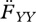 are the partial derivatives of *F* computed at *x*_0_, *y*_0_. The first two terms in (3.2) quantify the contribution to epistasis through individual traits *X* and *Y*, respectively, whereas the third term is associated with their functional coupling. As a generalized form of equation (3.1), equation (3.2) provides a direct quantitative link between the magnitude and direction of epistasis and the magnitude of individual mutations. For a function *F = X − Y*, upon expressing the *Δx* and *Δy* parameters in terms of *λ* and *θ*, it is easy to show that equation (3.2) yields the benefit–cost result of equation (3.1) (see electronic supplementary material, §D). One can further use equation (3.2) to explore expected epistasis under other possible functional dependencies. For example, if *F* is a linear combination of the two phenotypes, *F*(*X*,*Y*) = *aX* + *bY*, then the degree of epistasis converges to a generalized form of equation (3.1), where 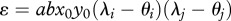. In this case, the relative signs of *a* and *b* determine whether the expected distribution of epistasis will display a positive or negative bias. In addition, as expected, one can verify that, if *F*(*X*,*Y*) = *XY*, then epistasis is always zero, compatible with the idea that multiplicative effects on individual traits will combine to provide an overall multiplicative effect on fitness. Interestingly, this is also true for functions of the form *F*(*X,Y*) *= X^n^Y^m^*, hinting to a broader view of the relationship between epistasis and independence, as explored in detail later.

An important question one can ask using the expression of equation (3.2) is what degree of epistasis should be expected between two mutations each affecting only one of the two traits (i.e. non-pleiotropic mutations; figure 1*b*). Does our model support the general intuition that mutations affecting independent modules in a biological system should have no epistasis? Equation (3.2) allows us to ask this question in a formal way. In our framework, lack of pleiotropy is expressed by assuming, for example, that allele *i* affects only trait *X*, and allele *j* affects only *Y* (i.e. *Δx_j_ =* *Δy_i_* = 0, equivalent to *λ_j_* = *θ_i_* = 1, in the benefit–cost model; figure 1*b*). From equation (3.2), one can see that under these conditions epistasis can be expressed as3.3
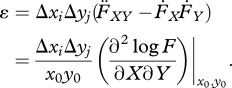
Equation (3.3) implies that, even if each mutation affects only one of the two phenotypes (figure 1*b*), epistasis will be zero only for the set of functions *F* that satisfy the condition ∂^2^log*F*/∂*x*∂*y =* 0. One can verify (see the electronic supplementary material, §D) that this condition is satisfied by any function decomposable as3.4
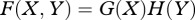


Note that, as opposed to functions such as *F*(*X,Y*) *= X^n^Y^m^*, which would always give zero epistasis (irrespective of whether mutations are pleiotropic or not), one can think of several other *F* functions that would satisfy equation (3.4), but give zero epistasis only in the absence of pleiotropy (e.g. *F*(*X,Y*) *=* exp(*X*) exp(*Y*), see electronic supplementary material, §D).

The consequences of equations (3.2)–(3.4) can be summarized as follows: (i) in the general case (arbitrary *F*), irrespective of whether mutations affect individual or multiple traits, one cannot necessarily expect zero epistasis relative to fitness, even if no epistasis is assumed relative to each individual trait; (ii) when *F* can be decomposed as in equation (3.4), epistasis can exist only in the presence of pleiotropy, i.e. if each mutation affects more than one trait. Hence, equation (3.4) can be viewed as an operational definition of independence between two traits; and (iii) in particular cases (e.g. if *F* is simply the product of two traits), epistasis will be always zero, irrespective of pleiotropy (table 3).

**Table 3.** The general expression of epistasis with and without pleiotropy. Equation (3.2) can be rewritten as 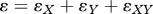 (first row), where 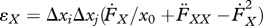, 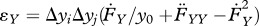 and 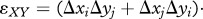 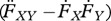. If each of the alleles *i* and *j* acts on a distinct trait with no pleiotropic effect (figure 1*b*; *Δx_j_* = *Δy_i_* = 0, or, equivalently, *Δx_i_* = *Δy_j_* = 0), then one obtains *ɛ_X_ = *ɛ*_Y_* = 0, and hence *ɛ* = *ɛ_XY_* . However, for any decomposable function *F*(*X*,*Y*) = *G*(*X*) · *H*(*Y*) (second row), *ɛ_XY_ =* 0 because 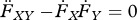. Therefore, when *F*(*X*,*Y*) = *G*(*X*) · *H*(*Y*), epistasis is non-zero only in the presence of pleiotropy, i.e. if *ɛ_X_* and/or *ɛ_Y_* are different from zero. For the particular case *F*(*X*,*Y*) = *X^n^Y^m^* (third row), epistasis is always zero, no matter whether or not there is pleiotropy.

	pleiotropic case (figure 1*a*)	non-pleiotropic case (figure 1*b*)
general *F*(*X*,*Y*)	*ɛ = *ɛ*_X_ + *ɛ*_Y_ + *ɛ*_XY_*	*ɛ = *ɛ*_XY_*
*F*(*X*,*Y*) = *G*(*X*)·*H*(*Y*)	*ɛ = *ɛ*_X_ + *ɛ*_Y_*	*ɛ* = 0
*F*(*X*,*Y*) = *X^n^Y^m^*	*ɛ* = 0	*ɛ* = 0

## 4. Discussion

Genome-wide epistatic profiles of fitness have been used to study the functional organization of biological systems [10], suggesting the existence of functionally coherent modules, characterized by specific epistatic interaction network properties such as monochromaticity [9,40]. Yet the relationship between the modular organization of the cell and epistasis remains poorly understood. Inspired by the successful attempt to explain epistasis data with a simple benefit–cost model of microbial fitness [7], we sought to explore the broader implications of expressing fitness as a function of two quantitative traits. We found that epistasis, and specific biases in its distribution, could be a natural outcome of the dependence of fitness on multiple phenotypes. If we interpret different phenotypes as metrics associated with different subsystems, or modules, we can determine whether epistasis exists at the system-level, and what might be the bias in its distribution based on how two modules interact with each other to produce fitness. Thus, we suggest that our approach establishes a novel link between biological modularity, pleiotropy and epistasis.

The benefit–cost model, originally employed to explain a small number of interactions in a single evolutionary experiment, has been shown here to lead to a global bias in the distribution of epistasis, under a broad range of beneficial versus deleterious mutation frequencies. In particular, upon deviating from symmetry in the amount of beneficial and deleterious mutations, we predict an overall pattern of negative *ɛ*. A similar pattern was previously suggested to be informative in identifying physically interacting partners or gene pairs belonging to redundant, but parallel functional pathways [10]. Besides its relevance to functional genomics, a trend towards negative *ɛ* also plays an important role in evolutionary theories seeking to interpret origin and maintenance of sex and recombination [3,4]. On the other hand, for adaptation, negative *ɛ* indicates that beneficial alleles combine antagonistically and suggests a diminishing returns trend in the fitness improvements as more beneficial alleles are acquired, consistent with some previous experimental results and theoretical analyses [7,8,41–43]. Thus, based on simple assumptions about the functional dependence of fitness on multiple phenotypes and genetic perturbations, a single ‘symmetry breaking’ mechanism could provide a potential explanation for both the deceleration of adaptation upon accumulation of beneficial mutations, and the prevalence of synergistic interactions between deleterious mutations. In pondering the general relevance of the above results to biology, one cannot avoid asking whether and why a simple benefit–cost model should truly underlie trends of epistasis, potentially across different organisms and biological scales. Indeed, we do not expect that a benefit–cost model should be the common mechanism behind all observed epistasis trends (see counter-example in Chou *et al*. [44]). However, it is not inconceivable that under some circumstances, fitness could be effectively represented as the overall difference of two independently measurable terms, for example, in cases where most mutations are estimated to affect the abundance of different proteins in the cell. While direct experimental testing is beyond the scope of the current paper, it is worth mentioning that the quantitative prediction of negative epistasis bias in our benefit–cost model does not deviate much from the corresponding value observed for epistatic interactions between yeast deletion mutants [10] upon matching our model's deleterious/beneficial allele ratio to the 3 : 1 ratio present in the yeast data (see the electronic supplementary material, figure S8). Given that our model is only remotely related to the yeast deletion data, this result may be pure coincidence. However, it exemplifies how the result we are presenting could be tested against experimental data in the future.

Whether or not the negative bias we observe in our model is directly relevant for the discussion on the evolution of recombination remains to be seen. Experimental data have provided conflicting results [23,29,32,45], depending on the system used (yeast, bacteria, viruses), the methods involved (classical versus high throughput), the different criteria for selecting genes to be analysed (highly deleterious only, YPD essential genes, etc.), and the definition of epistasis employed. In addition, while robust with respect to several parameters, the bias observed in the benefit–cost model may still in principle change in magnitude or sign, under different assumptions on the underlying distribution of individual mutations (see also electronic supplementary material, figures S2–S6). In any case, similar to prior computational models [9], we believe that the framework we are proposing will be useful in explaining, and potentially motivating experimental measurements relevant for this question.

Beyond the benefit–cost model, we showed in equation (3.2) that it is possible to estimate how epistasis depends on the magnitude of individual mutations and on the functional dependence of fitness on the two traits *X* and *Y*. For simple functional dependencies, this equation leads to direct insight into the type of epistasis to be expected. For example, linear combinations of traits lead to epistatic effects formally similar to the ones obtained with the benefit–cost model, except that the sign (and therefore the distribution biases) of epistasis is heavily influenced by the signs of the coefficients of such linear combinations. Future experiments (e.g. measuring epistasis upon perturbations of metabolic pathways that combine additively to produce fitness) could directly test this prediction. Our analytical expression is derived upon analysing the partial derivative of fitness with respect to traits it depends on, similar to the concept of ‘phenotype landscape’ proposed before to address the evolution of canalization, phenotypic plasticity and integration [46]. In our approach, however, we explicitly take into account both the effects of different mutations on distinct traits, and the functional dependence of fitness on such traits. One of the most interesting consequences of our general expression for epistasis as a function of two quantitative traits is the possibility to infer a general class of functional dependencies that guarantee lack of epistasis in the absence of pleiotropy. This finding echoes the viewpoint of a recent review that pleiotropy is an important prerequisite for epistasis [24]. Our result establishes a formal link between epistasis and pleiotropy, and suggests a new way to think about independence in biological networks.

Our method is simple and analytically solvable. Future variants of our framework could address more complex or alternative scenarios: first, given the multiplicity of traits that may be thought of as contributing to fitness, one could extend the current approach to fitness functions that depend upon more than two phenotypes (as we preliminarily explored here through computer simulations for the benefit–cost model). Second, while we have assumed here that perturbations correspond to genetic mutations, one could explore the consequences of a similar model for environmental perturbations. Third, it may be interesting to generalize our expression for epistasis to the case in which the basic traits themselves do have some degree of epistasis. In such case, one could seek an ‘epistasis propagation law’, showing how epistasis at a low-level phenotype affects epistasis at higher levels. Fourth, it has been suggested that the purging of deleterious mutations depends on the magnitude of mutational effects, in addition to the bias in the distribution of epistasis [23,47]. Future extension of our equation (3.2) in regard to linkage disequilibrium could further address this view point from another perspective to further elucidate this critical point about the evolution of sexual reproduction. Fifth, as we show in electronic supplementary material, figure S7, our model can account for instances of reciprocal sign epistasis between two alleles, providing potential new avenues for studying the ruggedness of evolutionary landscapes.

Finally, while in the current work we have focused on fitness and on its dependence on other traits, our formulation is quite general, and should equally apply to the functional dependence of any trait on any other set of traits. Even if most genetic interaction data are obtained relative to fitness, epistasis relative to multiple phenotypes will probably become increasingly available [31,48], offering opportunities to study how epistasis propagates between different traits to ultimately shape the genotype–phenotype mapping. We hope also that our equation will help understand epistasis relative to traits associated with genetic diseases, and provide insight on the interplay between evolution and modular organization of biological systems.
